# MicroRNA–Directed siRNA Biogenesis in *Caenorhabditis elegans*

**DOI:** 10.1371/journal.pgen.1000903

**Published:** 2010-04-08

**Authors:** Régis L. Corrêa, Florian A. Steiner, Eugene Berezikov, René F. Ketting

**Affiliations:** Hubrecht Institute, Royal Netherlands Academy of Arts and Sciences and University Medical Centre Utrecht, Utrecht, The Netherlands; Stanford University School of Medicine, United States of America

## Abstract

RNA interference (RNAi) is a post-transcriptional silencing process, triggered by double-stranded RNA (dsRNA), leading to the destabilization of homologous mRNAs. A distinction has been made between endogenous RNAi–related pathways and the exogenous RNAi pathway, the latter being essential for the experimental use of RNAi. Previous studies have shown that, in *Caenorhabditis elegans*, a complex containing the enzymes Dicer and the Argonaute RDE-1 process dsRNA. Dicer is responsible for cleaving dsRNA into short interfering RNAs (siRNAs) while RDE-1 acts as the siRNA acceptor. RDE-1 then guides a multi-protein complex to homologous targets to trigger mRNA destabilization. However, endogenous role(s) for RDE-1, if any, have remained unexplored. We here show that RDE-1 functions as a scavenger protein, taking up small RNA molecules from many different sources, including the microRNA (miRNA) pathway. This is in striking contrast to Argonaute proteins functioning directly in the miRNA pathway, ALG-1 and ALG-2: these proteins exclusively bind miRNAs. While playing no significant role in the biogenesis of the main pool of miRNAs, RDE-1 binds endogenous miRNAs and triggers RdRP activity on at least one perfectly matching, endogenous miRNA target. The resulting secondary siRNAs are taken up by a set of Argonaute proteins known to act as siRNA acceptors in exogenous RNAi, resulting in strong mRNA destabilization. Our results show that RDE-1 in an endogenous setting is actively screening the transcriptome using many different small RNAs, including miRNAs, as a guide, with implications for the evolution of transcripts with a potential to be recognized by Dicer.

## Author Summary

Due to its intrinsic characteristics, RNA interference (RNAi) has become one of the most widely used tools in cell biology and has revolutionized approaches to elucidate gene function. The process, also known as RNA silencing, is triggered by dsRNA molecules that are cleaved by Dicer proteins into small interfering RNAs (siRNAs). The *rde-1* gene from *Caenorhabditis elegans* was one of the first genes found in association with this mechanism and encodes the only Argonaute protein in worms, which is by itself essential for the classical RNAi pathway triggered by exogenously introduced dsRNA. However, little is known about endogenous functions of RDE-1. Here we show that RDE-1 binds to many classes of small RNAs, including microRNAs. We show that miR-243 is efficiently bound by RDE-1 and triggers regular RNAi on an endogenous target, implying that many RNA species, including miRNAs, are constantly being screened against the transcriptome using the canonical exogenous RNAi pathway.

## Introduction

RNA silencing is a conserved mechanism for controlling gene expression. In this process, long dsRNAs or hairpin-like transcripts are diced into small RNAs ranging from 21 to 26 nucleotides [1]. One of the strands of the small RNA duplex is retained in an RNA-induced silencing complex (RISC) to drive gene regulation at either post-transcriptional or transcriptional levels. The specificity is provided by the small RNA bound to RISC, and Argonaute proteins play an important role in this process [2]. Argonaute proteins are characterized by the presence of conserved PAZ and PIWI domains. The PAZ domain has been shown to be an RNA binding module, while the PIWI domain folds into an RNase H-like structure, which in some Argonaute proteins is catalytically active [3]. Argonaute proteins initially bind to small RNA duplexes, followed by removal of one of the duplex strands, known as the passenger strand. This leaves a single-stranded guide RNA bound by the Argonaute, ready to select homologous targets [4]. Argonaute proteins having RNase H activity can silence target-RNAs by endonucleolytic cleavage, but translation inhibition is also a very common mechanism of silencing.

The proliferation and diversification of silencing-related genes during evolution allowed the specialization of small RNA-directed pathways in controlling different biological fates [5]. Based on the available data so far, silencing pathways in the model nematode *Caenorhabditis elegans* can be schematically divided in two classes: endogenous, comprising microRNAs, 21U- RNAs, transposon repression and other chromatin-related processes, and exogenous, required for processing artificially introduced double-stranded RNA molecules (RNAi). *C. elegans* microRNAs are processed from endogenously expressed transcripts containing hairpin structures and are preferentially bound by the Argonaute proteins ALG-1 and ALG-2 [6]. From the other 25 Argonaute proteins found in *C. elegans*, RDE-1 is the only one required for processing long exogenous triggers. Although the PIWI domain of RDE-1 is catalytically active, we showed that its activity is only required for the maturation of the small RNA duplex and not for target RNA silencing [7].

In nematodes, plants and fungi, Argonaute proteins, together with RNA-dependent RNA polymerases (RdRP), are also involved in siRNA amplification [7]. When bound to primary, dsRNA-derived siRNAs, RDE-1 can recruit RdRPs that will use the targeted RNA as a template to generate secondary siRNAs, reinforcing the degradation process. There are four RdRPs in *C. elegans*, EGO-1 and RRF-1–3. EGO-1 and RRF-1 are key players in exo-RNAi pathways in germline and somatic tissues, respectively [8],[9]. So far, no clear function has been attributed to RRF-2, but RRF-3 is required for the accumulation of several classes of endogenous siRNAs [8]. In contrast to primary siRNAs, secondary siRNAs in *C. elegans* are only of antisense polarity relative to the target, have tri-phosphate groups at their 5′ ends [9]–[12], and are found mainly upstream of the primary recognition site. These features of secondary siRNAs in *C. elegans* have led to the suggestion they are direct products of RdRPs, instead of being Dicer products as observed in plants and fungi [13]. Once made, secondary siRNAs bind to a group of *C. elegans*-specific Argonaute proteins that will mediate further silencing. Indeed, knocking out multiple of these so-called secondary Argonaute proteins simultaneously leads to RNAi resistance [14].

Thus, it is clear that exo-RNAi occurs sequentially in *C. elegans*, and that RDE-1 is responsible for recruiting the amplification machinery in that scenario. But so far, no endogenous function has been attributed to RDE-1. Loading of small RNAs into RDE-1 depends largely on the structure of the duplex precursor. Fully matching precursor stems are preferentially loaded in RDE-1, but stems with 1-, 2- or 3-nucleotide mismatches are recognized as microRNAs and loaded into ALG-1 or ALG-2 [15]. However, it is not clear to what extent RDE-1 is loaded with such perfectly matching Dicer products *in vivo*, or whether RDE-1 is able to interact with other endogenous Dicer products as well.

We show that RDE-1 is loaded with a wide variety of Dicer products, and that miRNAs constitute a major fraction of the RDE-1-bound RNAs. The miRNA-RDE-1 complexes are functional and relevant, as we show that the gene Y47H10A.5 is silenced by miR-243 in an RDE-1- and RRF-1-dependent manner. Effectively, this makes RDE-1 a scavenger that surveys the complete *C. elegans* transcriptome for homology to any Dicer product, with implications for the evolution of any RNA molecule that may trigger Dicer activity.

## Results

### RDE-1 binds to many classes of small RNAs

**Figure 1:**
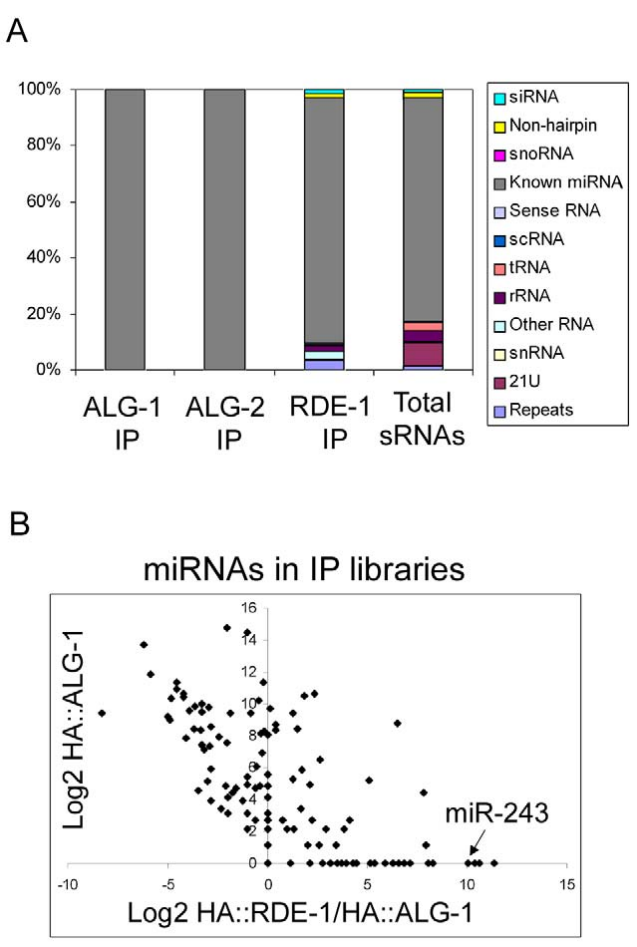
Overview of the small RNAs found in HA::ALG-1, HA::ALG-2, and HA::RDE-1 immunoprecipitate libraries. (A) Composition of small RNAs found in HA::ALG-1, HA::ALG-2, and HA::RDE-1 immunoprecipitates compared to those found in a library made of total small RNAs [18]. Small RNAs were divided in the twelve classes listed beside the graph. (B) A subset of miRNAs is enriched in HA::RDE-1 immunoprecipitates. In order to visualize the enrichment or depletion of miRNAs bound by RDE-1 versus ALG-1 as a function of total miRNA abundance (as defined by the abundance in ALG-1), the Log2 of the ratio of miRNA reads found in the HA::RDE-1 and HA::ALG-1 immunoprecipitate libraries was plotted against the Log2 of reads found in the HA::ALG-1 library. MiRNAs plotted left of the Y axis are depleted on RDE-1 immunoprecipitates, while those on the right are enriched in RDE-1.

Argonaute protein function is in many cases reflected by the set of small RNAs it binds to. In an approach to analyze the endogenous role of RDE-1, its associated small RNAs were cloned and sequenced. For that, RDE-1 complexes were purified from a mixed-stage *rde-1* mutant population, rescued with an HA-tagged RDE-1 construct [16]. RNAs were extracted from the purified RDE-1 complexes and were used to generate a small RNA library that was analyzed by massively parallel sequencing on a 454 platform (Figure 1A and Table S1). For comparison, small RNAs associated with ALG-1 and ALG-2, the two Argonaute proteins associated with miRNAs in *C. elegans*, were also cloned from mixed cultures of previously established HA-tagged lines (Figure 1A and Table S1) [17]. As all three Argonaute proteins have been shown to bind directly to Dicer products and to act upstream, or not linked to RdRP activity, RNA samples were not treated with phosphatase enzymes to allow cloning of RNA molecules carrying a 5′ tri-phosphate terminus. In our analysis we also included a previously published mixed-stage total RNA library [18]. We should note that, while also not treated with phosphatases to trim potential 5′-tri-phosphate groups, this wild-type total RNA library was constructed using a different cloning method that potentially affects cloning frequencies of certain small RNA molecules. One example of such a small RNA population could be 21U-RNAs, since these contain a 2′OMe modification at their 3′ end, which likely has a differential effect on RNA ligase, used by Ruby et al [18] and terminal transferase enzymes, as used in our study. Still, when assessing small RNA populations, and not individual sequences, a comparison between these libraries should be meaningful.

RDE-1, ALG-1 and ALG-2 small RNAs were predominantly 22 nt in length (data not shown). The small RNA reads were mapped to the *C. elegans* genome and divided into twelve categories: siRNA (antisense to coding regions), repeat RNA, known microRNA, 21U-RNA, scRNA (small cytoplasmic RNA), snRNA (small nuclear RNA), snoRNA (small nucleolar RNA), non-hairpin region, tRNA, rRNA, sense RNA and “other”. “Other” includes RNA species not falling into any of the 11 categories and contains sequences derived from non-genic, non-annotated regions.

As expected, ALG-1 and ALG-2 specifically bind miRNAs (Figure 1A and Table S1). Other classes of small RNAs were retrieved at background frequencies from ALG-1 and ALG-2 immunoprecipitates, indicating that ALG-1/2 binding is a good criterion for microRNA definition in *C. elegans*.

In sharp contrast to ALG-1 and ALG-2, RDE-1 associates with significant numbers of reads derived from many different classes (Figure 1A, Table S1). When compared to a total RNA library [18], reads coming from repetitive loci are enriched in RDE-1, whereas 21U-RNAs are depleted, indicating that they poorly interact with RDE-1 (Figure 1A, Table S1). Surprisingly however, microRNAs are still by far the predominant small RNA class associated with RDE-1 (Figure 1A, Table S1). Relative abundance of individual miRNA species varies between the three Argonaute proteins, with some miRNAs clearly enriched in RDE-1 immunoprecipitates (Figure 1B and Table S2).

### Absence of RDE-1 affects siRNAs, but not microRNAs

To further assess the role of RDE-1 in endogenous RNA silencing pathways, total small RNAs from *rde-1* mutant worms were cloned and deep-sequenced using an Illumina platform. To allow detection of indirect effects on secondary siRNAs carrying 5′-tri-phosphate groups, *rde-1* mutant RNA was treated with Tobacco Acid Pyrophosphatase (TAP). For comparison, we sequenced two wild-type libraries: one with TAP treatment and one without (Table S3).

**Figure 2:**
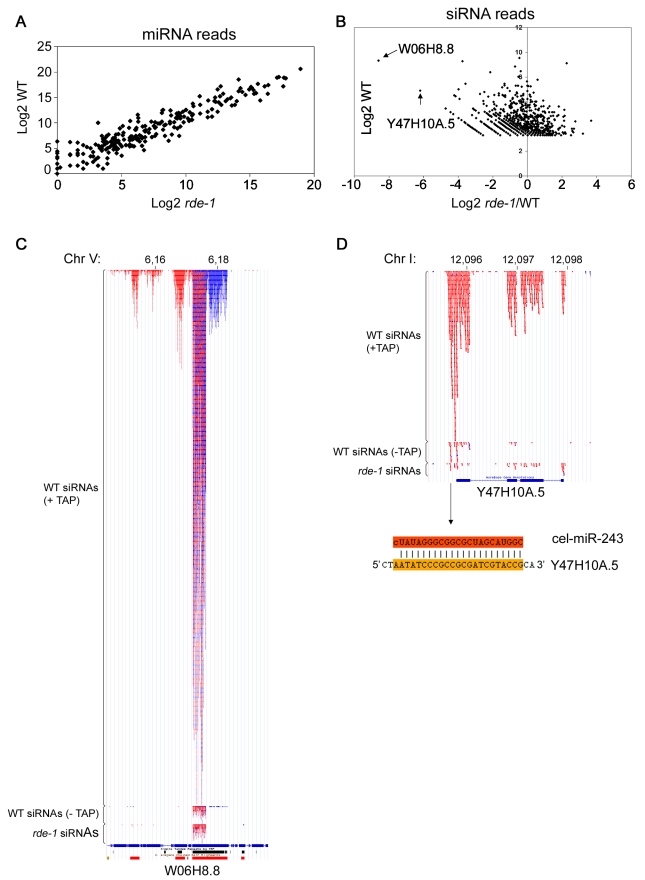
SiRNAs but not miRNAs are affected on *rde-1* mutant worms. Total small RNAs from N2 and *rde-1* worms were cloned and deep-sequenced. (A) Plot with the Log2 number of miRNA reads found in N2 and *rde-1* worms. Most miRNAs are equally found in N2 or *rde-1* libraries, indicating that absence of RDE-1 do not disturb their biogenesis or accumulation. (B) In order to visualize siRNA abundance in *rde-1* mutants relative to wild-type as a function of total siRNA abundance in wild-type animals, the Log2 of the ratio of siRNA reads found in the *rde-1* and N2 libraries was plotted against the Log2 of siRNA reads found in the N2 library. Dots left to the Y axis represent siRNAs affected by the *rde-1* mutation. The two most affected loci, W06H8.8 and Y47H10A.5, are pointed out with arrows. (C) Genomic view of siRNAs mapping to the W06H8.8 locus. Red and blue dots represent antisense and sense siRNAs, respectively. Part of W06H8.8 genomic structure is represented below the siRNA dots. Blue boxes are exons and lines represent introns. Black and red boxes below the genomic structure represent simple tandem repeats and self alignments, respectively. (D) Genomic view of siRNAs mapping to the Y47H10A.5 locus. Color coding as in (C). The miR-243 binding site is shown below the Y47H10A.5's genomic structure. The upper sequence is miR-243 and the lower sequence the 3′ UTR of the Y47H10A.5 transcript.

When the wild-type and *rde-1* mutant libraries were compared, no significant differences in microRNA levels were observed (Figure 2A). Therefore, although RDE-1 binds to many miRNAs, it likely interacts with only a minor fraction of them, and, as expected, is not required for their biogenesis or accumulation. However, when levels of siRNAs are compared between the two libraries, some noticeable differences are observed (Figure 2B). Two prominent siRNA loci are particularly affected in *rde-1* mutant animals, W06H8.8 and Y47H10A.5 (hereafter indicated as W06 and Y47, respectively), with both loci producing significantly less siRNAs in the *rde-1* mutant.

### W06 siRNAs

W06H8.8 encodes at least six protein isoforms that are orthologs of human TITIN, a giant muscle protein expressed in cardiac and skeletal tissues [19]. Like the majority of endo-siRNAs in *C. elegans*, W06 siRNAs depend strongly on TAP treatment for efficient cloning, indicating that they are predominantly secondary siRNAs (Figure 2C). Therefore, the effect of *rde-1* on W06 siRNAs is likely indirect.

W06 siRNAs are most abundant in a core region of the W06H8.8 transcript where approximately equal amounts of sense and antisense siRNAs are found. This core siRNA region is flanked by regions producing mostly sense siRNAs on the 5′ side and mostly antisense siRNAs on the 3′ side (Figure 2C). This constellation suggests that the core region produces some dsRNA structures that are the primary trigger for the observed secondary siRNA production. Indeed, the central siRNA region folds into several hairpin-like structures, suggesting that RNA-RNA pairing might be implicated in their biogenesis (data not shown). In addition, the existence of secondary siRNAs derived from both genomic strands implies that the region is covered by two overlapping transcripts that may contribute to the production of dsRNA and thus primary siRNA formation. Unfortunately, we could not detect coverage of the core W06 region in the RDE-1 IP library, most likely due to the fact that most of the RDE-1-associated RNAs represent miRNAs, thus making the siRNA coverage of individual loci too low in our medium scale sequencing effort of the RDE-1 IP library. Thus, at present the complexity of the locus prevents a full dissection of cause and consequence of siRNA production.

### Y47 siRNAs

Y47H10A.5 is a gene encoding a small protein with unknown function (Figure 2D). It is a member of a gene family containing six copies (Figure S1). All of these are located in each other's close vicinity, suggesting that they were formed through recent duplications. Functionally, nothing is known about these proteins. Knock-down experiments using RNAi have not revealed phenotypes [20]–[22], potentially because of their multi-copy nature. In order to place our results that will be described below in a biological context, we determined the expression pattern of Y47H10A.5 using a transgene containing an upstream Y47H10A.5 fragment driving GFP. As depicted in Figure S2, this revealed that Y47H10A.5 is strongly and exclusively expressed in intestinal cells.

The siRNAs coming from the Y47 locus are fully dependent on 5′ tri-phosphate trimming, designating them as secondary siRNAs. Y47 siRNAs are strictly antisense to the Y47H10A.5 gene, and are restricted to exons, suggesting that they are made by an RdRP enzyme using spliced transcripts from this gene as a template. Furthermore, siRNAs are more abundant on the 3′ end of the transcript, progressively declining towards the 5′ end, suggesting that RdRP recruitment takes place at the 3′ end of the transcript (Figure 2D). In fact, we observed that most siRNA-producing loci have higher siRNA coverage on their 3′ part compared to their 5′ part (data not shown). Yet, the Y47 locus is the only locus (along with W06) strongly affected in *rde-1* mutants. We set out to investigate the reason behind this unique feature.

### MiR-243 is a potential initiator of Y47 siRNA production

**Figure 3:**
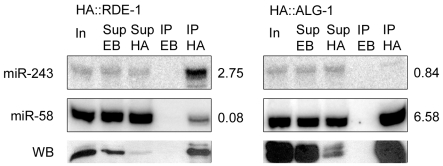
Mir-243 is enriched in HA::RDE-1 immunoprecipitates. RNAs from input extract (In), empty-beads supernatant (Sup EB), HA-beads supernatant (Sup HA), empty-beads immunoprecipitates (IP EB), or HA immunoprecipitates (IP HA) from HA::RDE-1 or HA::ALG-1 worms were extracted and checked by northern blot for the presence of miR-243 or miR-58. The ratio between the amount of RNA in the IP HA and Sup EB fractions is indicated beside blots. Proteins from the same fractions were also extracted and checked by western blots (WB) using HA-tag specific antibodies. Equal amounts of input extract or RNA and supernatant extract or RNA were loaded.

An inspection of the Y47H10A.5 sequence on miRBase revealed the presence of two microRNA binding sites within the 3′ UTR, one for miR-1 and one for miR-243. As shown in Figure 2D, miR-243 has full complementarity to its Y47H10A.5 binding site. Furthermore, miR-243 precursor RNA has extensive base pairing in the stem [23], making it a good RDE-1 co-factor, as already suggested by the frequent cloning of miR-243 from RDE-1 immunoprecipitates (Figure 1B; Table S2). As observed for Y47H10A.5 (Figure S2), promoter-GFP fusions showed that miR-243 is also highly expressed in intestinal cells [24]. We therefore wondered whether Y47 siRNAs could be made through an endo-RNAi mechanism triggered by an RDE-1::miR-243 complex. First, we confirmed the apparent enrichment for miR-243 in RDE-1, as seen by sequencing (Figure 1B; Table S2), using Northern blot analysis. We found that miR-243 is indeed enriched in RDE-1, but not in ALG-1 immunoprecipitates (Figure 3). As expected, when membranes were probed for miR-58, a microRNA derived from a mismatched precursor, the results were inverted: efficient precipitation was observed with ALG-1, but only weak with RDE-1 (Figure 3).

### Genetic requirements for RDE-1::miR-243 triggered siRNAs

**Figure 4:**
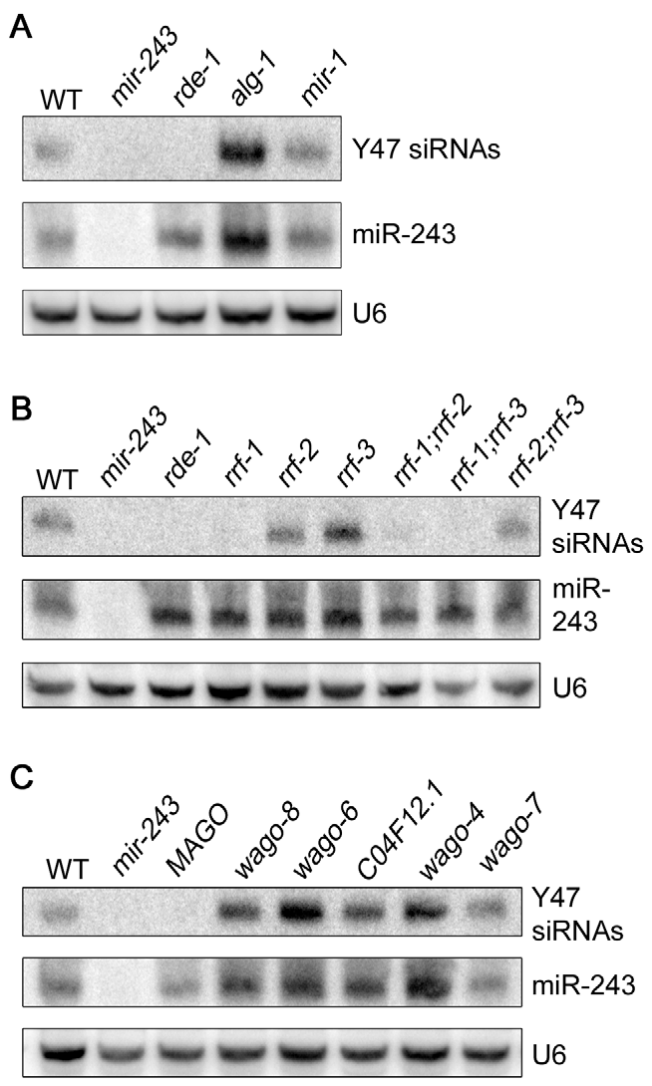
Mir-243 triggers a full endo-RNAi reaction on the Y47H10A.5 locus. The small RNA fraction from different worm lines was extracted and analyzed by northern blot by using probes against Y47H10A.5 siRNAs (Y47 siRNAs) or miR-243. U6 was used as loading control. (A) Primary Argonaute and miRNA mutants, (B) RdRP mutants and (C) secondary Argonaute mutants were tested for Y47 siRNA production.

We checked the genetic requirements for the generation of Y47 siRNAs by using Northern blot. Y47 siRNAs were readily detected in wild-type animals, and they were still present in *alg-1* or *mir-1* mutant worms (Figure 4A). In contrast, Y47 siRNAs were completely gone in *rde-1* or *mir-243* mutants (Figure 4A). These results show that miR-243 is required for the production of Y47 siRNAs and that RDE-1, and not ALG-1, is the involved Argonaute protein. Interestingly, levels of both miR-243 and Y47 siRNAs were slightly elevated in *alg-1* mutants, indicating that the RDE-1- and ALG-1-mediated pathways are not fully separated, but may compete for small RNA co-factors.

In the *C. elegans* exo-RNAi pathway, secondary siRNAs are made by RdRP enzymes and then loaded into secondary Argonaute proteins, also called WAGOs. We asked whether accumulation of Y47 siRNAs requires a similar machinery. Indeed, Y47 siRNAs are greatly reduced in *rrf-1*, *rrf-1;rrf-2* or *rrf-1;rrf-3* mutants (Figure 4B). They accumulate normally in *rrf-2*, *rrf-3* or *rrf-2;rrf-3* worms, indicating that RRF-1 is the predominant RdRP involved in their production. Single mutant lines for the Argonaute proteins *wago-4*, *wago-6*, *wago-7*, *wago-8* (also known as *F58G1.1*, *sago-2*, *ppw-1* and *sago-1*, respectively [25]) or *C04F12.1* had no effect on the accumulation of Y47 siRNAs. However, as observed for the exo-RNAi pathway [14], Y47 siRNAs are severely affected in the MAGO strain, a strain carrying multiple Argonaute mutant alleles simultaneously (Figure 4C). Together, our results show that miR-243, when loaded into RDE-1, can trigger the production of siRNAs and that the mechanism has similar genetic requirements to the exo-RNAi pathway.

### Y47 siRNAs trigger mRNA destabilization

**Figure 5:**
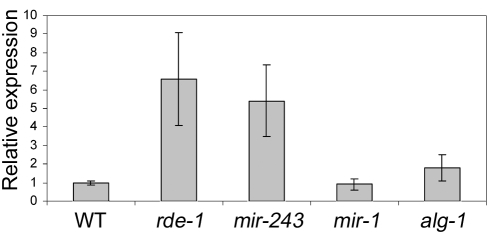
Y47 transcript is upregulated in RNAi– or miR-243–deficient worms. The relative expression ratios were measured by real time PCR and are the average of three biological replicates, using four different genes as controls (c*dc-42*, *eIF-3*, *Pmp-3*, and *Tba-1*).

To check if Y47 siRNAs are able to silence their target sequence, Y47H10A.5 transcript levels were analyzed by quantitative real-time PCR. Our results show that Y47 mRNA is upregulated in *rde-1* mutant animals relative to wild type (Figure 5), in agreement with previously published microarray experiments [26]. A similar level of upregulation was observed in *mir-243* mutant worms (Figure 5). Although miR-243 is relatively depleted in ALG-1 immunoprecipitates (Figure 3), a mild level of upregulation was also detected in *alg-1* mutant worms, while deletion of *mir-1*, the second miRNA targeting Y47H10A.5, had no effect on Y47 transcript levels (Figure 5). These results indicate that the ALG-1::miR-243 complex also slightly destabilizes the Y47H10A.5 transcript.

Most Y47 siRNAs map only once in the *C. elegans* genome and therefore are likely to act only on that locus. However, some of them also match to other genomic regions. To check whether Y47 siRNAs can act *in trans*, the expression of a transcript hit by one of them was analyzed by real-time PCR. As shown in Figure S3, transcript levels from Y47H10A.3, one of the Y47H10A.5 paralogs, are not affected in *mir-243* mutants, indicating that a single hit by a secondary siRNA is not sufficient to trigger mRNA decay. This makes a trans-acting mechanism for the Y47 siRNAs unlikely.

### Additional miR-243 targets

**Figure 6:**
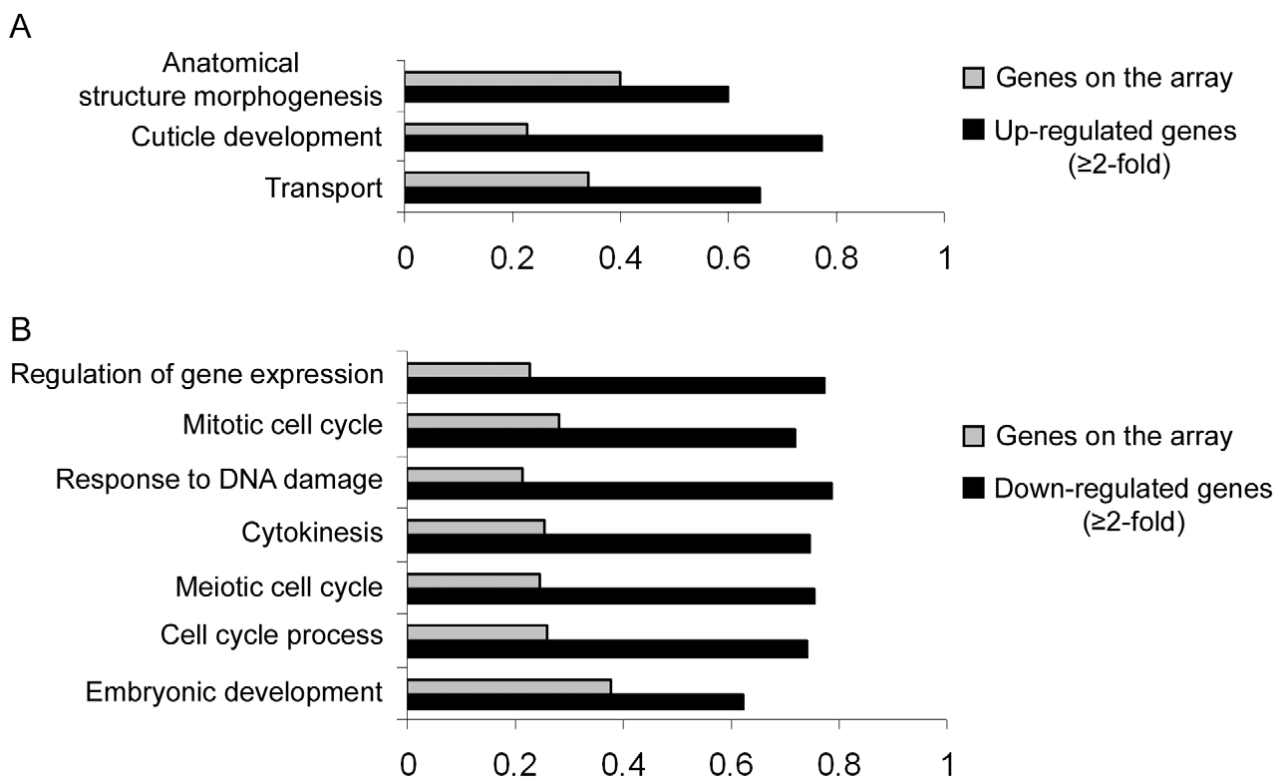
Direct and indirect roles of miR-243. RNA extracted from wild-type and *mir-243* adult worms were analyzed by microarray experiments. GO terms significantly overrepresented in the list of misregulated genes (black bars, fold-change ≥2) were compared to the total genes present on the array (gray bars). Bar size indicates the proportion of genes having the specified GO term. Genes found to be upregulated (A) or downregulated (B) in *mir-243* worms were considered separately. Only genes misregulated in three independent experiments were analyzed. The complete list of genes misregulated on the array can be found in Table S4.

Our results so far implicate miR-243 in an RNAi-like circuitry that is rather unusual for miRNAs. Prediction programs, however, indicate that miR-243 can potentially target other genes for silencing, and therefore could also act as a canonical microRNA. To probe for direct or indirect roles of miR-243, we performed microarray analysis comparing adult wild-type and mutant worms. Around 19% of the genes present on the array were misregulated in *mir-243* worms (fold change ≥2). From those, 48.7% were upregulated and 51.3% were downregulated (Table S4). As expected, the Y47H10A.5 gene was upregulated in the *mir-243* mutant background. Gene ontology analysis showed that terms involved with anatomical morphogenesis or transport are enriched in the list of upregulated genes (Figure 6A). In contrast, genes downregulated in *mir-243* worms are mainly involved with cell-cycle-related processes (Figure 6B). Using the MEME software [27] we asked whether specific miRNA seed sequences were overrepresented in the upregulated gene set. No specific miRNA signatures were revealed this way, preventing the identification of additional direct miR-243 targets. However, these results do indicate that miR-243 is an active gene with profound effects on gene expression.

## Discussion

### RDE-1 is loaded with many different small RNAs

In general, Argonaute proteins are loaded with specific subsets of small RNAs. Consistent with this idea, we find that ALG-1 is almost exclusively loaded with miRNAs (Table S1). In contrast, RDE-1 is loaded with different types of small RNAs, including many different miRNAs (Table S1 and S2). It was already known that RDE-1 can bind to miRNAs, specially when processed from precursors with limited mismatches in the stem [15], but the abundance and diversity of miRNAs found in RDE-1 immunoprecipitates was unexpected. However, when total small RNAs from *rde-1* worms were analyzed, microRNAs were largely unaffected, indicating that the protein is not required for their biogenesis (Figure 2A). Since RDE-1 forms a complex with Dicer [28], we propose that the protein probes small RNAs coming from many different Dicer-mediated pathways, and is effectively scanning the transcriptome for perfectly matching targets of the various Dicer products. This is consistent with a role in the defense against dsRNA-producing pathogens, as observed for RNAi in plants [29].

### A tasiRNA-like pathway in animals

Despite the many different short RNAs represented in RDE-1 immunoprecipitates, we found that not many loci are affected by loss of *rde-1*, at least in terms of secondary siRNA production (Figure 2A and 2B). Only two loci producing significant amounts of siRNAs in wild-type animals are severely affected by loss of *rde-1*. In case of the first locus, W06H8.8, many secondary siRNAs appear to be triggered by two converging transcripts. In this scenario it is difficult to discriminate the primary trigger and the subsequent silencing cascade, and we have at present not studied this locus further. At the second locus, Y47H10A.5, however, we could show that one individual miRNA is triggering secondary siRNA production. This miRNA, miR-243, binds to RDE-1 that in turn recognizes a perfectly matching sequence in the 3′UTR of the Y47H10A.5 mRNA. Following this, RRF-1 comes in to start synthesis of secondary siRNAs using the Y47 mRNA as a template. These siRNAs are then bound by other Argonaute proteins (WAGOs) that are likely responsible for the observed Y47 mRNA destabilization triggered by miR-243.

In plants, miRNAs can induce the production of siRNAs through the trans-acting siRNA (tasiRNAs) pathway. In that system, miRNA-guided cleavage of non-coding precursor transcripts (TAS transcripts) triggers the production of dsRNAs by RDR6, a plant RdRP. The resulting TAS-dsRNAs are subsequently cleaved by DICER-LIKE -4 into phased 21 nt-size tasiRNAs [30],[31]. Here we show that *C. elegans* miR-243 is also able to trigger the generation of siRNAs reminiscent of plant tasiRNAs (Figure 4). As in plants, miRNA-induced siRNAs are derived from an RNA silencing amplification process. In this process, RdRPs are recruited to the miRNA-targeted transcripts to generate several siRNAs. However, unlike plant tasiRNAs, Y47 siRNAs are only of antisense polarity, indicating that they are not Dicer products (Figure 2D). Instead, they are only dependent on the Argonaute RDE-1, on the RdRP RRF-1 and on secondary Argonautes (Figure 4), demonstrating that their production has all the characteristics of the well described exo-RNAi pathway in *C. elegans*. As Y47 siRNAs also do not act *in trans*, we conclude that the miR-243-Y47 interaction should not be seen as an animal version of the plant tasiRNA pathway.

### A role for RDE-1 in miRNA evolution?

Two mechanisms have been proposed for the birth of miRNAs: inverted duplication of genic sequences [32]–[34], and random selection of sequences producing hairpin-like transcripts [35],[36]. As RDE-1 appears to be generally loaded with Dicer products, any sequence evolving into a miRNA, or into any other RNA species having suitable double stranded RNA character, will be subject to a selection process that involves processing by Dicer, and subsequent RDE-1-mediated scanning for perfectly matching targets to the resulting short RNA. Interestingly, miR-243 is not conserved in other nematode species like *C. briggsae*, while many microRNA genes have well conserved homologs in *C. briggsae* and some even up to mammals. Also, miR-243 does not have a typical bulged miRNA precursor structure [23], and is loaded poorly into ALG-1 (Figure 3). Thus, the interaction between miR-243 and the Y47 locus may represent a case of an emerging miRNA, going through the above described selection process. Yet, miR-243 does have predicted canonical miRNA-like targets. However, our microarray analysis failed to detect a miR-243 seed signature in upregulated genes. As seed signatures are easily recognized in mammalian cell culture systems [37], this may indicate a lack of miR-243 activity. On the other hand, this can very well be related to the fact that, in our study, complete animals were analyzed, rather than pure cell lines, likely resulting in a much lower signal-to-noise ratio. Still, our microarray experiments indicate that miR-243 might have direct or indirect roles in many processes, most notably morphogenesis, transportation and cell-cycle.

The vast majority of miRNAs in *C. elegans*, and in any other animal system, do not have perfectly matching target mRNAs. Apparently, miRNAs that match perfectly to their targets are not tolerated well in animals. RDE-1 may provide a way to quickly recognize such perfect matches, followed by a silencing response that is much stronger than that typically triggered by a miRNA, ensuring that harmful interactions will be selected against firmly.

### Note added during the production process

While this work was under review, Gu et al [25] also showed that Y47 siRNAs require RDE-1, RRF-1, and secondary Argonautes for their accumulation.

## Materials and Methods

### Nematode strains

Nematodes were cultured according to standard procedures and the Bristol strain N2 was used as the standard wild-type strain. The alleles used in this study were *alg-1(gk214)*, *rde-1(pk3301)*, *rde-1(ne300)* rescued with *rde-1::HA* [16], *alg-1::HA(pkIs2250)* [17], *mir-243(n4759)*; *mir-1(gk276)*; *rrf-1(pk1417)*; *rrf-2(pk2040)*; *rrf-3(pk1426)*. The MAGO(WM126) strain has the following composition: *wago-8(tm1195); wago-6(tm0894); C04F12.1(tm1637); wago-4(tm1019); wago-7(tm0914)*. Note that *wago-6* is also known as *sago-2*, *wago-7* as *ppw-1*, *wago-8* as *sago-1* and *wago-4* as F58G1.1 [25].

### RNA–immunoprecipitation

Nematode extracts were obtained by freezing about 2 ml of nematodes in IP buffer (10 mM Tris-HCl (pH 7.5), 100 mM KCl, 2 mM MgCl2, 0.05% (v/v) Tween-20, 15% (v/v) glycerol, 5 mM DTT and 1 tablet of complete mini–EDTA-free protease inhibitor cocktail (Roche) per 10 ml buffer) in liquid nitrogen, followed by grinding with mortar and pestle and homogenizing with 20 strokes in a tight fitting dounce. Debris were spun down twice five minutes in a tabletop centrifuge at 10,000 g at 4°C.

For RNA immunoprecipitations, HA::ALG-1, HA::ALG-2 and HA::RDE-1 were immunoprecipitated from nematode extract by incubation with anti-HA 3F10 affinity matrix (Roche) for 2 h at 4°C. Protein G–agarose (Roche) was used as the ‘empty-beads’ control. After five washes with IP buffer, 1% of the immunoprecipitate was used to detect HA–RDE-1, HA-ALG-2 and HA–ALG-1 by western blotting according to standard procedures using anti-HA 3F10 (Roche).

RNA from nematode extracts and RNA-immunoprecipitates were isolated using Trizol LS (Invitrogen) according to the manufacturer's protocol. To assess the quality of the immunoprecipitated RNA, 1% of the RNA was treated with shrimp alkaline phosphatase (Promega) and ^32^P-5′-labeled using T4 poly-kinase (Promega). The remaining RNA was used for library construction or northern blot analysis.

### Library construction and massively parallel sequencing

For IP libraries, the small-RNA cDNA library was prepared by Vertis Biotechnology AG (Freising-Weihenstephan, Germany) to be suitable for massively parallel sequencing using 454 technology as described previously [38]. Briefly, the small RNA fraction was enriched by excision of the 15 to 30 nt fraction from a polyacrylamide gel. For cDNA synthesis, the RNA molecules in this fraction were first poly A-tailed using poly(A)polymerase followed by ligation of synthetic RNA adapter to the 5′ phosphate of the miRNAs. First strand cDNA synthesis was then performed using an oligo(dT)-linker primer and M-MLV-RNase H- reverse transcriptase. cDNA was PCR-amplified with adapter-specific primers and used in single-molecule sequencing. Massively parallel sequencing was performed by MWG Biotech AG (Ebersberg, Germany) using the Genome Sequencer 20 system.

For mutant libraries, worms were staged as young adults, lysed by Protease K treatment and RNA was isolated using Trizol (Invitrogen). The RNAs were, or were not treated with Tobacco Acid Pyrophosphatase and subsequently poly(A)-tailed using poly(A) polymerase followed by ligation of a RNA adaptor to the 5′-phosphate of the small RNAs. First-strand cDNA synthesis was performed using an oligo(dT)-linker primer and M-MLV-RNase H- reverse transcriptase. The cDNA was PCR-amplified for 16 cycles according to the instructions of Illumina/Solexa. Equal amounts of bar-coded cDNA were mixed. The 125–140 bp fraction of the cDNA pool was obtained by fractionation of the cDNA on a preparative 6% polyacrylamide (PAA) gel. The eluted cDNA was finally purified using the Macherey & Nagel NucleoSpin Extract II kit and was sent for sequencing on an Illumina/Solexa platform.

All sRNA sequences obtained in this work has been deposited in the GEO database with the accession number GSE20649.

### Sequence analysis

Computational analysis of small RNA reads was performed as described previously [38]. Adaptor sequences were trimmed from generated data using custom scripts. Resulting inserts were mapped to the *C. elegans* genome (ce4) using the megablast program [39], and genomic annotations of mapped reads were retrieved from the Ensembl database (release 52, dec 2008) using Perl API provided by Ensembl [40]. Twelve classes were defined: siRNA, repeat RNA, known microRNA, 21U-RNA, scRNA (small cytoplasmatic RNA), snRNA (small nuclear RNA), snoRNA (small nucleolar RNA), non-hairpin region, tRNA, rRNA, sense RNA and other. The “other” class includes RNA species derived from non-genic, non-annotated regions.

### Northen blot analysis

For detection of small RNAs, total RNA fraction extracted from young-adult worms was enriched for small RNAs using Mirvana kit (Ambion) and 10 µg were fractionated on 15% polyacrylamide gels and transferred to Hybond-N membrane (Roche). Pre-hybridization/hybridization and washes were done at 32°C for siRNAs and 37°C for microRNAs in ULTRAhyb-oligo hybridization buffer (Ambion). For detection of miRNAs, oligonucleotides corresponding to the antisense sequences of the small RNAs were synthesized and radioactively end-labeled with [α-^32^P]ATP by using T4 polynucleotide kinase (Promega). Probes used in this study were: miR-243 – 5′ ATATCCCGCCGCGATCGTACCG 3′; miR-58 – 5′ TTGCCGTCTGCGTCTC 3′; Y47H10A.5 siRNAs: 5′ GTTTCAGTTCTGGGGAATTTGGTCTAAAAAACTAT 3′ and 5′ TCACTATTTGGCCAGTTAATCTTATATTTCCCGG 3′;

### Real-time PCR

For RT reactions, 5 µg of total DNase-treated RNAs were reverse-transcribed with M-MLV (Promega) and oligo dT primers. Quantitative RT-PCR was carried out using the MyiQ Single-Color Real-Time PCR Detection System from Bio-Rad. Each reaction contained: 10 µl of the iQ SYBR Green qPCR SuperMix (Biorad), 0.2 µM of forward and reverse primers and 5 µl of cDNA (1∶25 RNA dilution) in a total volume of 20 µl. PCR cycles were as follows: one cycle of 95°C for 3 min; 40 cycles of 95°C for 15 sec; 60°C for 30 sec and 72°C for 45 sec. Results were normalized against *Cdc-42*, *eIF-3*, *Pmp-3* and *Tba-1*, four housekeeping genes known to be stable in *C. elegans* [41]. Each sample was run in triplicate and a mean value of each Ct triplicate was used for further calculation through the 2^−ΔCt^ method. Results presented are the average values obtained from three biological replicates. Primers used were: Y47 Forward (5′ TCGGGATAGTGAAATGAAAGC 3′), Y47 Reverse (5′ GGTTGTTCGTCTTGTAGGCAA 3′), Cdc-42 forward (5′ ATGTCCGAGAAAAATGGGTG 3′), Cdc-42 Reverse (5′ ATCCGTTGACACTGGTTTCTG 3′), eIF-3 Forward (5′ GCTGAGACTGTTAAGGGAATGG 3′), eIF-3 Reverse (5′ GAGCGAAACAGTGGCATAAAC 3′), Pmp-3 Forward (5′ GGAATTGTTTCACGGAATGC 3′), Pmp-3 Reverse (5′ TGGAGGACGATCAGTTTCAA 3′), Tba-1 Forward (5′ GTACACTCCACTGATCTCTGCTGACAAG 3′) and Tba-1 Reverse (5′ CTCTGTACAAGAGGCAAACAGCCATG 3′).

### Microarrays

RNA samples from wild-type and *mir-243* young-adult worms were isolated and reverse-transcribed with random primers. Each cDNA sample was split and labeled with both Cy3 and Cy5. Samples were hybridized to Agilent *C. elegans* whole genome arrays at 65°C overnight. Signals were analyzed with the Agilent Array assist Software, utilizing background subtraction, LOWESS normalization, and dyeswap analysis. RNAs that changed at least 2-fold with a probability of p<0.05 in three biological replicates were considered differentially regulated between wild-type and *mir-243*.

Gene ontology analysis was carried out by the online software FatiGO (http://babelomics.bioinfo.cipf.es/). FatiGO software provides different levels of GO analysis. Low levels consider only the general function of the genes. The higher the level, the more specific it becomes. Misregulated genes were analyzed in all available levels, including all three GO classifications (biological process, cellular component and molecular function). But only results for level 4 (p≤0.01) are shown.

The microarray data reported here has been deposited in the GEO database under the accession number GSE20558.

## Supporting Information

Figure S1 Phylogenetic analysis of the Y47H10A.5 sequence and its paralogs. (A) Phylogenetic tree showing the relatedness of Y47H10A.5 with its paralogs. The predicted amino acid sequences were aligned with Multalign and the tree was constructed by the neighbor-joining method. Bootstrap values are indicated at each node. (B) Percentage identity between the amino acid sequences of Y47H10A.5 and its related sequences. (C) Multiple sequence alignments of the deduced amino acid sequences. Black boxes represent residues that are present in all sequences in the alignment. Columns in the alignment with less than 100% conservation but more than 60% are shaded in grey. Shading was performed by GeneDoc program (http://www.psc.edu/biomed/genedoc/).
(1.33 MB TIF)

Figure S2 Expression pattern of Y47H10A.5. An approximately 1kb region upstream of the Y47H10A.5 sequence was cloned behind GFP and injected into wild-type worms. Expression is confined to intestine cells.
(2.56 MB TIF)

Figure S3 Y47 siRNAs do not act *in trans*. Level of Y47H10A.3 transcript was compared between wild type and *mir-243* worms by real-time PCR. The relative expression ratios are the average of three biological replicates, using three different genes as controls (*Cdc-42*, *eIF-3*, and *Pmp-3*).
(0.15 MB TIF)

Table S1 Overview of small RNAs found in the immunoprecipitate libraries of ALG-1, ALG-2, and RDE-1compared to those found in the library made of total small RNAs (From Ruby et al 2006 [18]).
(0.03 MB DOC)

Table S2 Cloning frequencies of microRNAs found in the libraries made from ALG-1, ALG-2, and RDE-1 immunoprecipitates and in the library from total small RNAs (Ruby et al 2006 [18]). Libraries are scaled to 100,000 reads for comparison. Note that the individual IP libraries had lower read coverage (Table S1).
(0.16 MB DOC)

Table S3 Overview of small RNAs found in the libraries of wild-type (with or without TAP treatment) and *rde-1* mutant animals.
(0.04 MB DOC)

Table S4 List of genes misregulated in *mir-243* worms.
(0.67 MB XLS)
