# Supplementary figures and images for: MicroRNA–Directed siRNA Biogenesis in Caenorhabditis elegans
Source: PLoS Genet. 2010 Apr 8;6(4):e1000903. doi: 10.1371/journal.pgen.1000903 (PMC2851571; doi:10.1371/journal.pgen.1000903)

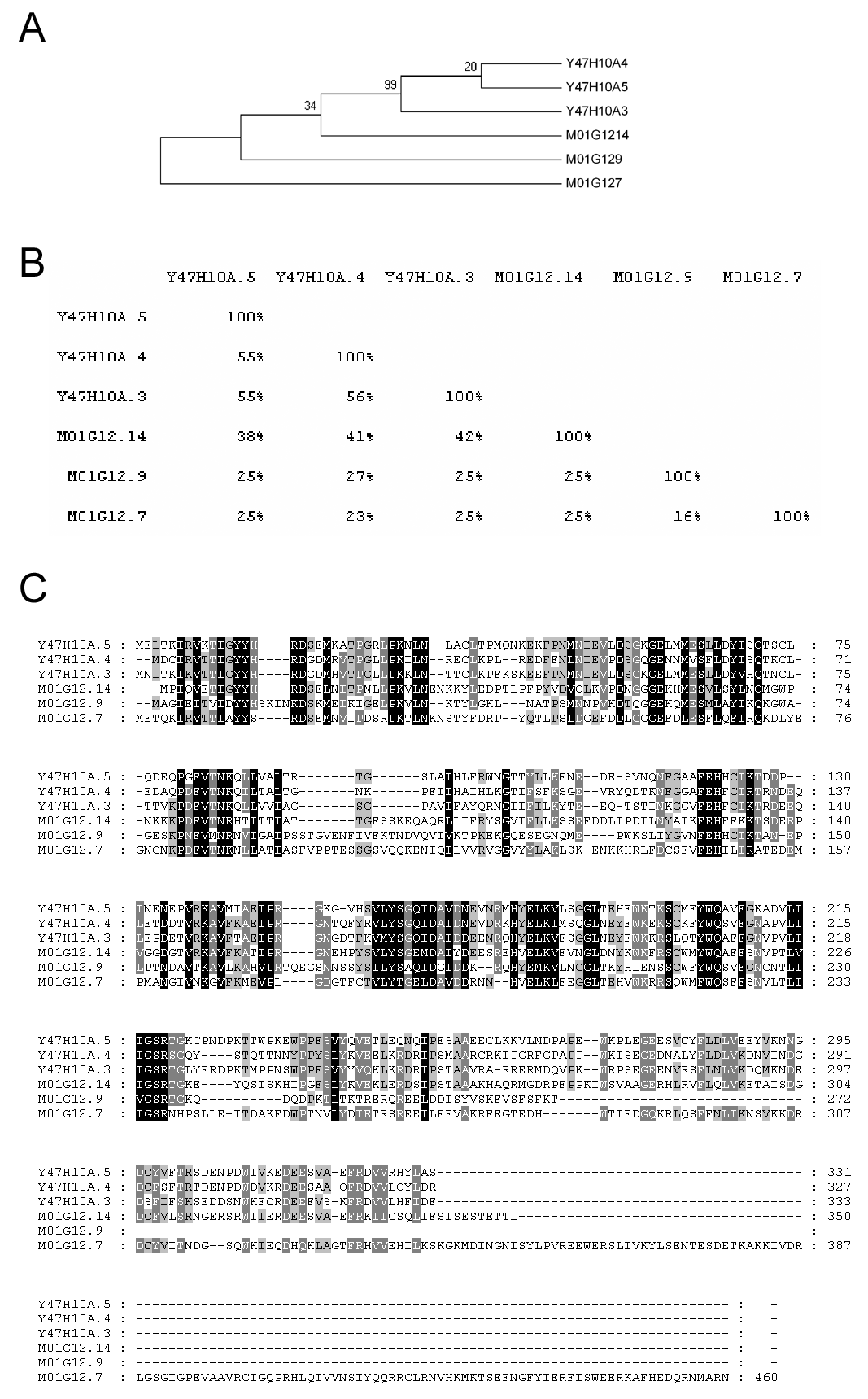

Supplement: Figure S1 — Phylogenetic analysis of the Y47H10A.5 sequence and its paralogs. (A) Phylogenetic tree showing the relatedness of Y47H10A.5 with its paralogs. The predicted amino acid sequences were aligned with Multalign and the tree was constructed by the neighbor-joining method. Bootstrap values are indicated at each node. (B) Percentage identity between the amino acid sequences of Y47H10A.5 and its related sequences. (C) Multiple sequence alignments of the deduced amino acid sequences. Black boxes represent residues that are present in all sequences in the alignment. Columns in the alignment with less than 100% conservation but more than 60% are shaded in grey. Shading was performed by GeneDoc program (http://www.psc.edu/biomed/genedoc/). (1.33 MB TIF) [file pgen.1000903.s001.tif]

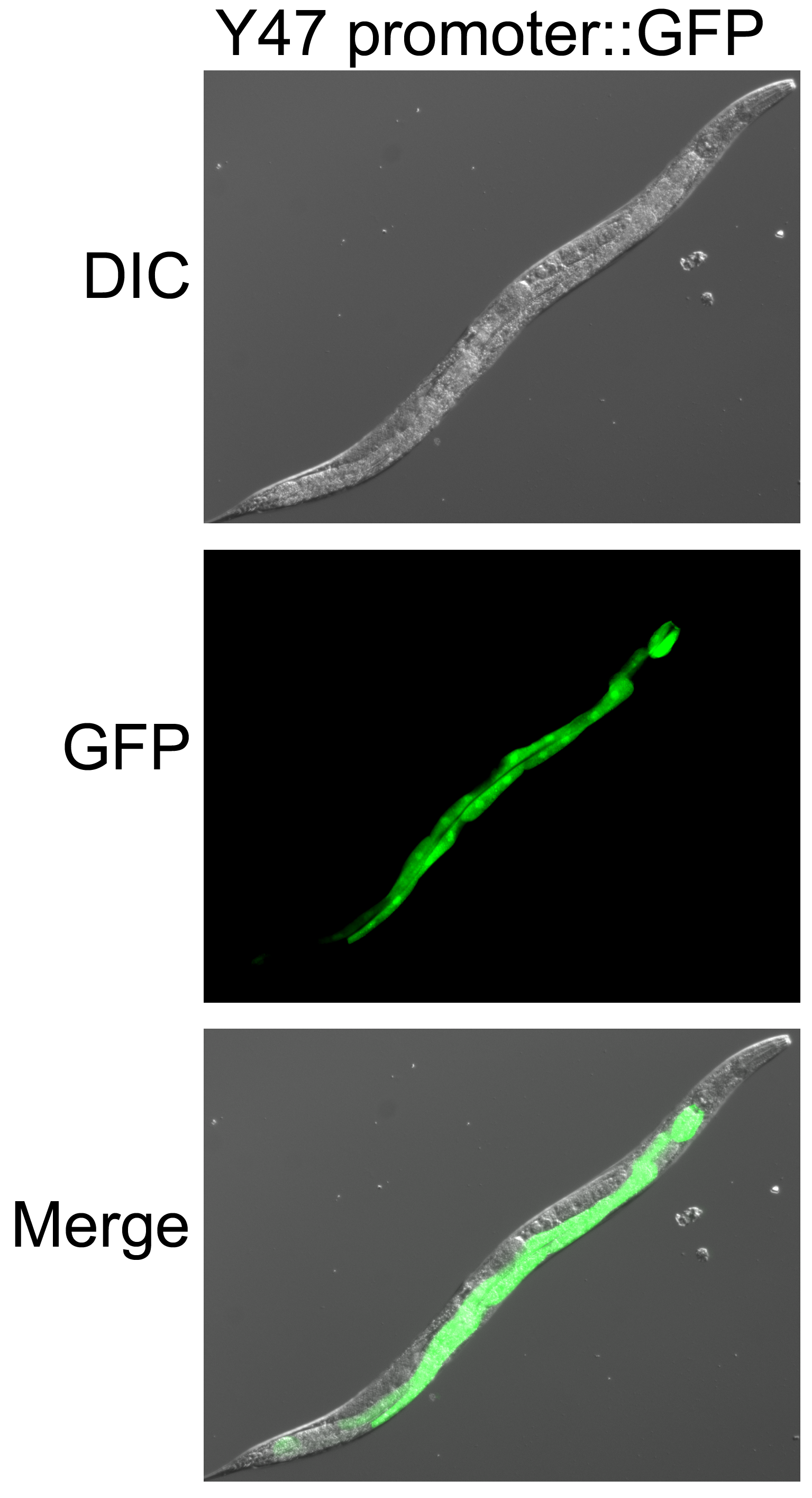

Supplement: Figure S2 — Expression pattern of Y47H10A.5. An approximately 1kb region upstream of the Y47H10A.5 sequence was cloned behind GFP and injected into wild-type worms. Expression is confined to intestine cells. (2.56 MB TIF) [file pgen.1000903.s002.tif]

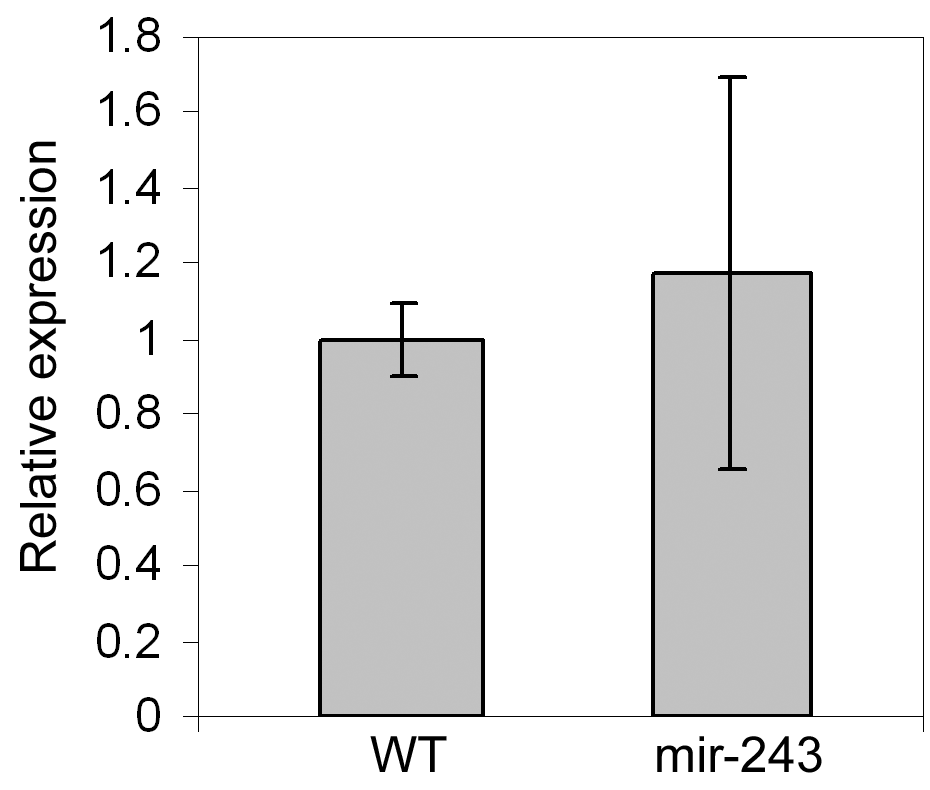

Supplement: Figure S3 — Y47 siRNAs do not act in trans. Level of Y47H10A.3 transcript was compared between wild type and mir-243 worms by real-time PCR. The relative expression ratios are the average of three biological replicates, using three different genes as controls (Cdc-42, eIF-3, and Pmp-3). (0.15 MB TIF) [file pgen.1000903.s003.tif]
