# Supplementary material for: MicroRNA–Directed siRNA Biogenesis in Caenorhabditis elegans
Source: PLoS Genet. 2010 Apr 8;6(4):e1000903. doi: 10.1371/journal.pgen.1000903 (PMC2851571; doi:10.1371/journal.pgen.1000903)
Supplement: Table S1 — Overview of small RNAs found in the immunoprecipitate libraries of ALG-1, ALG-2, and RDE-1compared to those found in the library made of total small RNAs (From Ruby et al 2006 [18]). (0.03 MB DOC) [file pgen.1000903.s004.doc]

**Table S1**. Overview of small RNAs found in the immunoprecipitate libraries of ALG-1, ALG-2 and RDE-1compared to those found in the library made of total small RNAs (From Ruby et al 2006).

| **Category** | **ALG1 IP** | **ALG2 IP** | **RDE1 IP** | **Total sRNAs** |
| --- | --- | --- | --- | --- |
| Repeats | 12 | 1 | 1853 | 5188 |
| 21U | 5 | 0 | 17 | 37013 |
| snRNA | 0 | 0 | 19 | 329 |
| Other RNA | 1 | 0 | 1582 | 324 |
| rRNA | 0 | 0 | 1041 | 16309 |
| tRNA | 0 | 1 | 337 | 12411 |
| scRNA | 0 | 0 | 0 | 227 |
| senseRNA | 9 | 2 | 212 | 2678 |
| Known miRNA | 45439 | 5988 | 45142 | 339775 |
| snoRNA | 1 | 1 | 23 | 613 |
| non-hairpin | 26 | 2 | 858 | 7322 |
| siRNA | 8 | 1 | 638 | 4061 |
| **Total** | 45501 | 5996 | 51722 | 426250 |
