# Supplementary material for: MicroRNA–Directed siRNA Biogenesis in Caenorhabditis elegans
Source: PLoS Genet. 2010 Apr 8;6(4):e1000903. doi: 10.1371/journal.pgen.1000903 (PMC2851571; doi:10.1371/journal.pgen.1000903)
Supplement: Table S2 — Cloning frequencies of microRNAs found in the libraries made from ALG-1, ALG-2, and RDE-1 immunoprecipitates and in the library from total small RNAs (Ruby et al 2006 [18]). Libraries are scaled to 100,000 reads for comparison. Note that the individual IP libraries had lower read coverage (Table S1). (0.16 MB DOC) [file pgen.1000903.s005.doc]

**Table S2**. Cloning frequencies of microRNAs found in the libraries made from ALG-1, ALG-2 and RDE-1 immunoprecipitates and in the library from total small RNAs (Ruby et al 2006). Libraries are scaled to 100000 reads for comparison. Note that the individual IP libraries had lower read coverage (Table S1).

| **miRNA** | **ALG1IP** | **ALG2IP** | **RDE1IP** | **Total sRNAs** |
| --- | --- | --- | --- | --- |
| cel-mir-58 3arm | 23201.23 | 10956.61 | 11606.17 | 9847.095 |
| cel-mir-48 5arm | 863.0559 | 184.2855 | 110.7248 | 2235.52 |
| cel-mir-56 3arm | 920.2994 | 1641.816 | 75.29287 | 1655.633 |
| cel-mir-55 3arm | 523.9982 | 1993.634 | 17.71597 | 2315.689 |
| cel-mir-80 3arm | 13150.59 | 37828.78 | 181.5887 | 6201.794 |
| cel-mir-66 5arm | 704.5354 | 1424.024 | 194.8757 | 3491.2 |
| cel-lin-4 5arm | 708.9388 | 167.5322 | 73.07837 | 2062.415 |
| cel-mir-72 5arm | 336.856 | 536.1032 | 947.8043 | 2863.805 |
| cel-mir-81 3arm | 1405.768 | 3258.502 | 76.40012 | 2211.172 |
| cel-mir-90 3arm | 330.251 | 720.3887 | 33.21744 | 697.7649 |
| cel-mir-65 5arm | 1635.843 | 1021.947 | 88.57984 | 1521.424 |
| cel-mir-84 5arm | 11.00837 | 0 | 35.43194 | 798.4212 |
| cel-mir-52 5arm | 28747.25 | 9499.079 | 7130.677 | 11650.59 |
| cel-mir-1 3arm | 160.7221 | 234.5451 | 22.14496 | 4349.005 |
| cel-mir-46 3arm | 1041.391 | 184.2855 | 106.2958 | 992.0138 |
| cel-mir-82 3arm | 1940.775 | 4950.578 | 83.0436 | 1988.778 |
| cel-let-7 5arm | 39.63012 | 0 | 97.43783 | 1153.242 |
| cel-mir-57 5arm | 171.7305 | 100.5193 | 17.71597 | 1418.393 |
| cel-mir-64 5arm | 1327.609 | 2546.49 | 46.50442 | 1066.541 |
| cel-mir-51 5arm | 328.0493 | 452.3371 | 431.8267 | 824.2533 |
| cel-mir-71 5arm | 1424.483 | 569.6096 | 5134.309 | 2637.848 |
| cel-mir-238 3arm | 440.3347 | 703.6354 | 40857.45 | 133.0207 |
| cel-mir-228 5arm | 343.461 | 820.908 | 26.57395 | 362.5408 |
| cel-mir-54 3arm | 744.1656 | 1005.193 | 48.71891 | 2181.183 |
| cel-mir-47 3arm | 383.0911 | 0 | 53.14791 | 1034.177 |
| cel-mir-237 5arm | 121.092 | 83.76612 | 99.65232 | 169.542 |
| cel-mir-73 3arm | 28.62175 | 100.5193 | 6.643488 | 768.4321 |
| cel-mir-77 3arm | 675.9137 | 6416.485 | 1607.724 | 1417.502 |
| cel-mir-229 5arm | 2659.621 | 1289.998 | 2303.076 | 189.4357 |
| cel-mir-50 5arm | 281.8142 | 201.0387 | 225.8786 | 701.9218 |
| cel-mir-250 3arm | 189.3439 | 33.50645 | 46.50442 | 43.05358 |
| cel-mir-86 5arm | 2677.235 | 703.6354 | 117.3683 | 3694.294 |
| cel-mir-61 3arm | 61.64685 | 16.75322 | 8.857984 | 618.7838 |
| cel-mir-244 5arm | 30.82343 | 0 | 15.50147 | 167.1666 |
| cel-mir-70 3arm | 15.41171 | 67.0129 | 2.214496 | 930.5511 |
| cel-mir-59 3arm | 2.201673 | 0 | 8.857984 | 114.3147 |
| cel-mir-241 5arm | 11.00837 | 0 | 2.214496 | 521.0967 |
| cel-mir-60 3arm | 275.2092 | 921.4274 | 276.812 | 1265.775 |
| cel-mir-63 3arm | 28.62175 | 184.2855 | 22.14496 | 138.0684 |
| cel-mir-42 3arm | 68.25187 | 150.779 | 46.50442 | 436.1773 |
| cel-mir-45 3arm | 591.1493 | 360.1943 | 18.82322 | 1274.534 |
| cel-mir-44 3arm | 591.1493 | 360.1943 | 18.82322 | 1273.644 |
| cel-mir-2 3arm | 673.712 | 653.3758 | 376.4643 | 4434.222 |
| cel-mir-252 5arm | 226.7723 | 268.0516 | 13.28698 | 18.40912 |
| cel-mir-236 3arm | 140.9071 | 33.50645 | 15.50147 | 217.9402 |
| cel-mir-85 3arm | 416.1162 | 67.0129 | 558.053 | 722.7063 |
| cel-mir-53 5arm | 821.2241 | 435.5838 | 907.9434 | 627.3945 |
| cel-mir-80 5arm | 0 | 0 | 2.214496 | 23.7537 |
| cel-mir-62 3arm | 0 | 33.50645 | 310.0295 | 365.2131 |
| cel-mir-235 3arm | 3742.845 | 100.5193 | 66.43488 | 214.3771 |
| cel-mir-230 3arm | 28.62175 | 33.50645 | 0 | 8.313794 |
| cel-mir-74 3arm | 242.1841 | 201.0387 | 44.28992 | 2507.5 |
| cel-mir-87 3arm | 28.62175 | 167.5322 | 6.643488 | 25.2383 |
| cel-mir-34 5arm | 1215.324 | 150.779 | 901.2999 | 497.343 |
| cel-mir-35 3arm | 4.403347 | 636.6225 | 62.00589 | 638.6776 |
| cel-mir-234 3arm | 0 | 0 | 0 | 18.11219 |
| cel-mir-40 3arm | 312.6376 | 2998.827 | 281.241 | 832.5671 |
| cel-mir-259 5arm | 48.43681 | 234.5451 | 0 | 9.204558 |
| cel-mir-67 3arm | 92.47028 | 117.2726 | 586.8415 | 325.7226 |
| cel-mir-242 5arm | 15.41171 | 0 | 2.214496 | 25.53523 |
| cel-mir-39 3arm | 57.24351 | 1105.713 | 188.2322 | 684.1065 |
| cel-mir-83 3arm | 8.806693 | 16.75322 | 4.428992 | 1036.255 |
| cel-mir-76 3arm | 17.61339 | 67.0129 | 0 | 38.89668 |
| cel-mir-231 3arm | 35.22677 | 33.50645 | 4.428992 | 95.90556 |
| cel-mir-239a 5arm | 6.60502 | 0 | 112.9393 | 233.0832 |
| cel-mir-75 3arm | 26.42008 | 67.0129 | 8.857984 | 657.3836 |
| cel-mir-788 5arm | 2.201673 | 0 | 0 | 225.0663 |
| cel-mir-246 3arm | 37.42845 | 83.76612 | 1255.619 | 18.11219 |
| cel-mir-786 3arm | 0 | 16.75322 | 2.214496 | 24.94138 |
| cel-mir-233 3arm | 17.61339 | 33.50645 | 4.428992 | 108.3762 |
| cel-mir-787 3arm | 8.806693 | 16.75322 | 0 | 10.09532 |
| cel-mir-46 5arm | 8.806693 | 0 | 2.214496 | 31.77057 |
| cel-mir-71 3arm | 0 | 0 | 35.43194 | 96.4994 |
| cel-mir-793 3arm | 0 | 33.50645 | 115.1538 | 21.67525 |
| cel-mir-232 3arm | 689.1237 | 284.8048 | 2.214496 | 238.7247 |
| cel-mir-47 5arm | 4.403347 | 0 | 11.07248 | 44.53818 |
| cel-mir-43 3arm | 26.42008 | 33.50645 | 17.71597 | 875.0268 |
| cel-mir-253 3arm | 15.41171 | 0 | 6.643488 | 8.313794 |
| cel-mir-124 3arm | 8.806693 | 0 | 2.214496 | 933.5203 |
| cel-mir-36 3arm | 4.403347 | 167.5322 | 33.21744 | 1014.283 |
| cel-mir-357 3arm | 0 | 16.75322 | 0 | 2.969212 |
| cel-mir-795 5arm | 0 | 0 | 2.214496 | 1.187685 |
| cel-mir-1820 5arm | 0 | 0 | 15.50147 | 1.781527 |
| cel-mir-239a 3arm | 22.01673 | 0 | 5000.332 | 9.501479 |
| cel-mir-248 3arm | 0 | 16.75322 | 0 | 13.36146 |
| cel-mir-255 3arm | 0 | 0 | 0 | 2.672291 |
| cel-mir-49 3arm | 4.403347 | 33.50645 | 2.214496 | 60.86885 |
| cel-mir-243 3arm | 0 | 0 | 1044.135 | 29.09828 |
| cel-mir-799 3arm | 0 | 0 | 0 | 10.98609 |
| cel-mir-355 5arm | 6.60502 | 0 | 0 | 0.296921 |
| cel-mir-245 3arm | 22.01673 | 16.75322 | 6.643488 | 1.484606 |
| cel-mir-240 3arm | 44.03347 | 0 | 22.14496 | 24.94138 |
| cel-mir-58 5arm | 6.60502 | 0 | 11.07248 | 69.77649 |
| cel-mir-1022 5arm | 2.201673 | 16.75322 | 0 | 0.593842 |
| cel-mir-358 3arm | 0 | 0 | 59.79139 | 0.593842 |
| cel-mir-241 3arm | 2.201673 | 0 | 0 | 0.890764 |
| cel-mir-790 5arm | 0 | 16.75322 | 139.5133 | 5.344582 |
| cel-mir-1829c 5arm | 0 | 0 | 0 | 23.7537 |
| cel-mir-63 5arm | 2.201673 | 0 | 13.28698 | 18.70604 |
| cel-mir-38 3arm | 0 | 0 | 4.428992 | 448.351 |
| cel-mir-789 3arm | 0 | 0 | 2635.25 | 20.68551 |
| cel-mir-247 3arm | 2.201673 | 0 | 0 | 16.33067 |
| cel-mir-229 3arm | 0 | 0 | 6.643488 | 92.93634 |
| cel-mir-1829a 3arm | 0 | 0 | 0 | 5.047661 |
| cel-mir-240 5arm | 0 | 0 | 1330.912 | 4.75074 |
| cel-mir-77 5arm | 2.201673 | 0 | 0 | 2.37537 |
| cel-mir-788 3arm | 0 | 0 | 0 | 2.969212 |
| cel-mir-72 3arm | 0 | 0 | 0 | 1.484606 |
| cel-mir-785 3arm | 4.403347 | 0 | 0 | 4.156897 |
| cel-mir-84 3arm | 0 | 0 | 1559.005 | 6.235346 |
| cel-mir-230 5arm | 2.201673 | 0 | 0 | 1.187685 |
| cel-mir-1020 5arm | 0 | 0 | 2.214496 | 0.296921 |
| cel-mir-54 5arm | 8.806693 | 0 | 8.857984 | 15.73682 |
| cel-mir-66 3arm | 0 | 0 | 11.07248 | 4.75074 |
| cel-mir-1829a 5arm | 0 | 0 | 0 | 0.890764 |
| cel-mir-1020 3arm | 0 | 16.75322 | 0 | 0.593842 |
| cel-mir-60 5arm | 0 | 0 | 2.214496 | 2.37537 |
| cel-mir-250 5arm | 0 | 0 | 0 | 7.719952 |
| cel-mir-1817 5arm | 6.60502 | 0 | 0 | 28.20752 |
| cel-mir-41 3arm | 0 | 0 | 0 | 181.7158 |
| cel-mir-359 3arm | 2.201673 | 0 | 0 | 2.969212 |
| cel-lsy-6 3arm | 0 | 0 | 0 | 0 |
| cel-mir-243 5arm | 0 | 0 | 8.857984 | 3.859976 |
| cel-mir-260 3arm | 0 | 0 | 0 | 0.890764 |
| cel-let-7 3arm | 0 | 0 | 0 | 5.047661 |
| cel-mir-262 3arm | 0 | 0 | 0 | 0.890764 |
| cel-mir-48 3arm | 6.60502 | 0 | 31.00295 | 10.39224 |
| cel-mir-789 5arm | 0 | 0 | 6.643488 | 0.296921 |
| cel-mir-79_novel 5arm | 0 | 0 | 42.07543 | 1.187685 |
| cel-mir-61 5arm | 0 | 0 | 0 | 1.484606 |
| cel-mir-1821 3arm | 0 | 16.75322 | 0 | 0.296921 |
| cel-mir-1822 3arm | 0 | 0 | 0 | 5.641503 |
| cel-mir-75 5arm | 0 | 0 | 0 | 11.28301 |
| cel-mir-34 3arm | 30.82343 | 33.50645 | 132.8698 | 86.10715 |
| cel-mir-83 5arm | 0 | 0 | 0 | 3.563055 |
| cel-mir-791 3arm | 0 | 0 | 19.93046 | 0.296921 |
| cel-mir-52 3arm | 24.21841 | 0 | 2.214496 | 78.09028 |
| cel-mir-249 3arm | 0 | 0 | 2.214496 | 5.938424 |
| cel-mir-791 5arm | 0 | 0 | 22.14496 | 0.296921 |
| cel-mir-797 5arm | 4.403347 | 0 | 8.857984 | 4.156897 |
| cel-mir-232 5arm | 2.201673 | 0 | 544.766 | 3.266133 |
| cel-mir-358 5arm | 0 | 0 | 77.50736 | 0.593842 |
| cel-mir-254 3arm | 0 | 0 | 0 | 6.829188 |
| cel-mir-55 5arm | 0 | 0 | 2.214496 | 23.45678 |
| cel-mir-49 5arm | 0 | 0 | 0 | 18.40912 |
| cel-mir-1829b 5arm | 0 | 0 | 0 | 42.16281 |
| cel-mir-78 3arm | 0 | 0 | 0 | 62.9473 |
| cel-mir-1819 3arm | 0 | 0 | 0 | 2.672291 |
| cel-mir-2 5arm | 0 | 0 | 0 | 59.08732 |
| cel-mir-45 5arm | 0 | 0 | 0 | 21.97217 |
| cel-mir-237 3arm | 2.201673 | 0 | 24.35946 | 0 |
| cel-mir-792 3arm | 0 | 16.75322 | 0 | 1.187685 |
| cel-mir-1830 3arm | 0 | 0 | 4.428992 | 0.890764 |
| cel-mir-65 3arm | 0 | 16.75322 | 4.428992 | 37.11515 |
| cel-mir-1 5arm | 0 | 0 | 6.643488 | 42.75666 |
| cel-mir-43 5arm | 2.201673 | 0 | 0 | 0.593842 |
| cel-mir-90 5arm | 0 | 0 | 2.214496 | 15.4399 |
| cel-mir-2212 5arm | 0 | 0 | 0 | 0.890764 |
| cel-mir-70 5arm | 0 | 0 | 0 | 1.484606 |
| cel-mir-259 3arm | 0 | 0 | 0 | 12.17377 |
| cel-mir-794 3arm | 0 | 0 | 2.214496 | 0 |
| cel-mir-51 3arm | 0 | 0 | 13.28698 | 1.187685 |
| cel-mir-800 5arm | 0 | 0 | 95.22333 | 1.484606 |
| cel-mir-239b 5arm | 0 | 0 | 0 | 12.47069 |
| cel-mir-44 5arm | 0 | 0 | 0 | 16.03375 |
| cel-mir-53 3arm | 2.201673 | 0 | 0 | 43.05358 |
| cel-mir-2210 3arm | 0 | 0 | 0 | 0 |
| cel-mir-257 3arm | 0 | 0 | 0 | 0 |
| cel-mir-784 5arm | 0 | 0 | 0 | 3.266133 |
| cel-mir-1820 3arm | 0 | 0 | 0 | 0 |
| cel-mir-57 3arm | 0 | 0 | 2.214496 | 11.87685 |
| cel-mir-42 5arm | 0 | 0 | 0 | 4.453818 |
| cel-mir-242 3arm | 0 | 0 | 263.525 | 0.296921 |
| cel-mir-236 5arm | 6.60502 | 0 | 4.428992 | 0.296921 |
| cel-mir-794 5arm | 0 | 0 | 0 | 1.484606 |
| cel-mir-1830 5arm | 0 | 0 | 2.214496 | 2.078449 |
| cel-mir-41 5arm | 0 | 0 | 0 | 3.563055 |
| **Total** | 100000 | 100000 | 100000 | 100000 |
