# Supplementary material for: MicroRNA–Directed siRNA Biogenesis in Caenorhabditis elegans
Source: PLoS Genet. 2010 Apr 8;6(4):e1000903. doi: 10.1371/journal.pgen.1000903 (PMC2851571; doi:10.1371/journal.pgen.1000903)
Supplement: Table S3 — Overview of small RNAs found in the libraries of wild-type (with or without TAP treatment) and rde-1 mutant animals. (0.04 MB DOC) [file pgen.1000903.s006.doc]

**Table S3**. Overview of small RNAs found in the libraries of wild type (with or without TAP treatment) and *rde-1* mutant animals.

| **Category** | **WT (-)TAP** | **WT (+)TAP** | ***rde-1* (+)TAP** |
| --- | --- | --- | --- |
| Repeats | 16411 | 1074287 | 375648 |
| 21U | 113633 | 202755 | 83292 |
| snRNA | 812 | 8483 | 3215 |
| Other RNA | 1602 | 6197 | 3415 |
| rRNA | 45455 | 54552 | 31895 |
| tRNA | 7950 | 15246 | 6473 |
| scRNA | 1130 | 1389 | 744 |
| senseRNA | 7558 | 77937 | 34044 |
| Known miRNA | 5744090 | 6555168 | 2378186 |
| snoRNA | 2575 | 5151 | 2118 |
| non-hairpin | 44317 | 1913049 | 642673 |
| siRNA | 27513 | 1003181 | 409772 |
| **Total** | 6013046 | 10917395 | 3971475 |
